# Interleukin-6: evolving role in the management of neuropathic pain in neuroimmunological disorders

**DOI:** 10.1186/s41232-021-00184-5

**Published:** 2021-11-02

**Authors:** Kenichi Serizawa, Haruna Tomizawa-Shinohara, Shota Miyake, Kenji Yogo, Yoshihiro Matsumoto

**Affiliations:** 1 Product Research Department, Chugai Pharmaceutical Co., Ltd., 200, Kajiwara, Kamakura, Kanagawa 247-8530 Japan; 2 Research Planning Department, Chugai Pharmaceutical Co., Ltd., -135, Komakado, Gotemba, Shizuoka 412-8513 Japan

**Keywords:** Anti–IL-6 receptor antibody, Experimental autoimmune encephalomyelitis, IL-6, MR16-1, Multiple sclerosis, Neuromyelitis optica spectrum disorder, Neuropathic pain, Satralizumab

## Abstract

**Background:**

Neuropathic pain in neuroimmunological disorders refers to pain caused by a lesion or disease of the somatosensory system such as multiple sclerosis (MS) and neuromyelitis optica spectrum disorder (NMOSD). MS and NMOSD are autoimmune disorders of the central nervous system, and ≥ 50% of patients with these disorders experience chronic neuropathic pain. The currently available medications for the management of neuropathic pain have limited effectiveness in patients with MS and NMOSD, and there is an unmet medical need to identify novel therapies for the management of chronic neuropathic pain in these patients. In this review article, we summarize the role of interleukin-6 (IL-6) in the pathogenesis of MS and NMOSD and the ameliorative effects of anti–IL-6 therapies in mouse models of experimental autoimmune encephalomyelitis (EAE).

**Main body:**

Intraperitoneal injection of MR16-1, an anti–IL-6 receptor (IL-6R) antibody, reduced mechanical allodynia and spontaneous pain in EAE mice, which was attributed to a reduction in microglial activation and inhibition of the descending pain inhibitory system. The effect of anti–IL-6 therapies in ameliorating neuropathic pain in the clinical setting is controversial; a reduction in pain intensity has been reported with an anti–IL-6 antibody in four studies, namely a case report, a pilot study, a retrospective observational study, and a case series. Pain intensity was evaluated using a numerical rating scale (NRS), with a lower score indicating lesser pain. A reduction in the NRS score was reported in all four studies. However, in two randomized controlled trials of another anti–IL-6R antibody, the change in the visual analog scale pain score was not statistically significantly different when compared with placebo. This was attributed to the low mean pain score at baseline in both the trials and the concomitant use of medications for pain in one of the trials, which may have masked the effects of the anti–IL-6R antibody on neuropathic pain.

**Conclusion:**

Thus, anti–IL-6 therapies might have a potential to reduce neuropathic pain, but further investigations are warranted to clarify the effect of inhibition of IL-6 signaling on neuropathic pain associated with MS and NMOSD.

## Background

Multiple sclerosis (MS) is a chronic, inflammatory, and demyelinating autoimmune disease of the central nervous system (CNS) [1]. According to a nationwide study, the estimated crude prevalence of MS in Japan in 2003 was 7.7 (95% confidence interval [CI], 7.1–8.4) per 100,000 population, with an increasing trend over time [2]. The prevalence of neuropathic pain in patients with MS ranges from 50 to 86%, with the most common pain symptoms being ongoing dysesthetic pain in the lower extremities, paroxysmal pain (L’hermitte’s phenomenon and trigeminal neuralgia), and thermal and mechanical sensory abnormalities. Other forms of neuropathic pain, including migraine with or without aura and tension-type headache, are also more prevalent in patients with MS than in the general population [3].

Neuromyelitis optica (NMO) is a chronic, rare, inflammatory CNS disorder primarily manifesting as optic neuritis and longitudinally extensive transverse myelitis lesions and characterized by the presence of pathogenic autoantibodies (immunoglobulin G [IgG]) against aquaporin-4 (AQP4-IgG). Although typical NMO is AQP4-IgG seropositive, some patients who present with the clinical characteristics of NMO are AQP4-IgG seronegative, therefore, use of the term of neuromyelitis optica spectrum disorder (NMOSD) is advocated [4]. For the purpose of this review, the term NMOSD encompasses both NMO and NMOSD based on the international consensus diagnostic criteria for NMOSD [4]. The crude prevalence of NMOSD in northern Japan in 2016 was 4.1 (95% CI, 2.2–6.9) per 100,000 population [5]. Kanamori et al. reported that the prevalence of pain in patients with NMOSD was > 80% [6]. Furthermore, Asseyer et al. noted that neuropathic pain was present in around 80% of patients with AQP4-IgG seropositive as well as AQP4-IgG seronegative NMOSD [7]. Neuropathic pain in NMOSD can be permanent or intermittent such as Lhermitte’s phenomenon and is localized either on the extremities or on the trunk [8]. The pathogenesis of pain in NMOSD is unclear, but likely contributors include the spinal cord gray matter (since the lesions are centrally located), brainstem descending modulatory pathways, and astrocyte damage (since AQP4 water channels, the target of AQP4-IgG, are mainly expressed on astrocytic foot processes) [9].

Neuropathic pain refers to pain caused by a lesion or disease of the somatosensory system [10]. It represents a broad category of pain syndromes and is associated with a variety of central or peripheral neurological disorders [10]. Chronic neuropathic pain is associated with hyperalgesia (increased pain response to a normally painful stimulus) and allodynia (painful response to a normally innocuous stimulus) [11]. Neuropathic pain that develops secondary to demyelination, neuroinflammation, and axonal damage in the CNS is the most distressing and difficult type of pain to treat [12]. Patients with NMOSD frequently report pain in wide areas around the trunk and entire legs, whereas pain in patients with MS tends to be localized in the distal portions of the limbs [6]. As pain greatly affects the health-related quality of life of patients with MS and NMOSD [6], it is crucial to adequately control pain attacks. First-line treatment for neuropathic pain includes gabapentinoids (e.g., pregabalin and gabapentin), tricyclic antidepressants (TCAs) (e.g., amitriptyline, nortriptyline, and imipramine), and selective serotonin-norepinephrine reuptake inhibitors (SSNRIs) (e.g., duloxetine) [13, 14]. These medications act on calcium channels (pregabalin and gabapentin) or inhibit the reuptake of neurotransmitters such as norepinephrine and serotonin (TCAs and SSNRIs) [13]. However, the mechanisms underlying neuropathic pain in MS and NMOSD include activation of the microglia and impairment of the descending pain inhibitory system, which differ substantially from those of the other treatable causes of pain [12, 15]. Consequently, the conventional medications used for the management of neuropathic pain have limited effectiveness in patients with MS and NMOSD [8, 12]. In a retrospective, cross-sectional cohort study, 75.9% of patients with NMOSD and 37.8% of patients with MS received prescription pain medications; however, no patient with NMOSD and < 50% of patients with MS reported as being pain free despite taking these medications [16]. Therefore, there is an unmet medical need to identify novel treatment targets, which may lead to the development of novel therapies for the management of chronic neuropathic pain in patients with MS and NMOSD.

The immune response associated with damage in the CNS and peripheral nervous system may contribute to the development of neuropathic pain [17]. The endothelial damage and increased neuronal activity that occur at the site of nerve injury, the dorsal root ganglia, and the dorsal horn of the spinal cord result in the recruitment of monocytes/macrophages in the periphery and microglial activation centrally, which leads to the release of pro-inflammatory mediators causing sensitization of neurons, thereby enabling a positive feedback to sustain chronic pain [17]. Cytokines such as interleukin-6 (IL-6) are released by inflammatory cells such as macrophages and monocytes in response to tissue injury or disease and can directly sensitize nociceptors [18]. Therefore, neutralization of pro-inflammatory cytokines appears to be a promising strategy for the treatment of neuropathic pain, given the wide range of effects such as direct reduction of nociception, decrease in inflammation (which drives the nociceptive sensitization processes), and inhibitory impact on neuron–glia interactions [19].

Four studies, namely a case report, a pilot study, a retrospective observational study, and a case series have evaluated the efficacy of off-label use of a humanized IgG1 anti–IL-6 receptor (IL-6R) monoclonal antibody in patients with NMOSD [20–23]. Pain intensity was evaluated using a numerical rating scale (NRS), with a lower score indicating lesser pain [20–23]. In one case report, the NRS score decreased from 4 to 0 within four administrations of the anti–IL-6R antibody [20]. A reduction in the NRS score was reported in the other three studies as well [21–23] (Table 1). Satralizumab, a humanized IgG2 anti–IL-6R monoclonal recycling antibody, is indicated for the treatment of NMOSD in adult patients who are AQP4-IgG seropositive [26, 27]. The effect of satralizumab on neuropathic pain in patients with NMOSD was evaluated as a secondary endpoint in two phase 3, randomized, placebo-controlled trials [24, 25]. The adjusted mean between-group difference in the change from baseline in the visual analog scale score for pain was not significant, which was attributed to the low mean score for pain at baseline in both the trials and the concomitant use of medications for pain in one of the trials [25]. The results of these clinical trials on the analgesic effects of IL-6 signaling inhibition are controversial due to the differential settings among trials. Therefore, further investigations are warranted.

**Table 1:** Efficacy and safety of anti–IL-6 therapies on neuropathic pain in patients with NMOSD

Anti–IL-6 therapy						
Author, year (study design)	Clinical characteristics of patients	Prior/concomitant medications	Dose, frequency of administration, and duration of treatment	Efficacy (NRS/VAS)		Safety
				NRS/VAS at baseline	NRS/VAS at the end of treatment/last follow-up	
Araki et al., 2013 (CR) [20]	A 36-year-old female patient with NMO	Combination of PSL and AZA	8 mg/kg i.v. every month for 6 months	NRS: 4	NRS: 0	Decline in SBP, lymphocytopenia, viral enteritis, and upper respiratory infection of unknown origin
Araki et al., 2014 (pilot study) [21]	Seven (six female and one male) patients with refractory AQP4-Ab-seropositive NMO or NMOSD	PSL or AZA alone or PSL in combination with AZA, CyA, or tacrolimus	8 mg/kg every month for 1 year	NRS, mean ± SEM: 3.0 ± 1.5	NRS at 6 months, mean ± SEM: 1.3 ± 1.3 NRS at 12 months, mean ± SEM: 0.9 ± 1.2	Upper respiratory infection (*n* = 2), acute enterocolitis (*n* = 2), acute pyelonephritis (*n* = 1), leukocytopenia, and/or lymphocytopenia (*n* = 3), anemia (*n* = 2), and a slight decline in SBP (*n* = 1)
Ringelstein et al., 2015 (ROS) [22]	Eight female patients with highly active AQP4-Ab-seropositive NMO (*n* = 6) or NMOSD (*n* = 2)	Immunomodulatory or immunosuppressant therapy (e.g., rituximab, interferon beta-1β, AZA)	6–8 mg/kg at a 4- to 6-week interval followed up to 10–51 months	NRS, median ((IQR): 6.5 (5.0–7.0)	NRS, median (IQR): 2.5 (0.3–4.5) (*p* = 0.02) 7/8 patients had less pain at the last follow-up, with two of them completely pain free	Mild post infusion nausea (*n* = 1), transient gastritis (*n* = 1), transient diarrhea (*n* = 1), headache (*n* = 1), fatigue (*n* = 2), recurrent urinary tract infections (*n* = 3), deep venous thrombosis (*n* = 1), transient mild liver enzyme increase (*n* = 3), recurrent CRP elevation (*n* = 1), leukopenia or neutropenia (*n* = 2) and elevation of cholesterol levels (*n* = 6)
Araki, 2019 (CS) [23]	19 patients with refractory NMOSD	Corticosteroids and/or immunosuppressants	Dose not specified; monthly infusion up to 6 years and 8 months	NRS, mean ± SD: 3.2 ± 2.2	NRS at 1 year after treatment: 1.7 ± 2.6 (*p* < 0.001) In one patient with comorbid SLE, severe neuropathic pain disappeared	Not reported
Yamamura et al., 2019 (CT) [24]	83 patients with AQP4-Ab- seropositive or AQP4-Ab- seronegative NMOSD: satralizumab, 41; placebo, 42	Oral glucocorticoids, AZA, MMF, AZA + glucocorticoids, and MMF+oral glucocorticoids	120 mg s.c. at weeks 0, 2, and 4 and every 4 weeks during the double-blind period^a^	VAS (mean ± SD Satralizumab group: 27.6 ± 28.2 Placebo group: 34.6 ± 26.1	The between-group difference in the change in the mean VAS pain score was 4.08 (95% CI, − 8.44 to 16.61, *p* = 0.52)	Satralizumab vs placebo (%): Infection (68% vs 62%), IRR (12% vs 5%), neoplasm^b^ (7% vs 7%), and serious infection (5% vs 7%)
Traboulsee et al, 2020 (CT) [25]	95 patients with AQP4-Ab- seropositive or AQP4-Ab-seronegative NMOSD: satralizumab,63; placebo, 32	Previous: B-cell depleting therapy and immunosuppressants. Analgesics were permitted during satralizumab therapy	120 mg s.c. at weeks 0, 2, and 4 and every 4 weeks thereafter in the double-blind period (maximal duration of 1.5 years after the random assignment of the last patient enrolled)	VAS (mean ± SD) Satralizumab group: 31.7 ± 28.9 Placebo group: 27.6 ± 30.8	The adjusted mean of the VAS pain score change from baseline did not differ significantly between the two groups (between-group difference in mean score change 3.21 (95% CI, − 5.09 to 11.52; *p* = 0.44).	Satralizumab vs placebo (%): Infections (54% vs 44%), IRR (13% vs 16%), and serious infections (10% vs 9%)

^a^The duration of the double-blind period ended when the total number of relapses reached 26. The median duration of treatment was 107.4 weeks (range, 2 to 224) in the satralizumab group and 32.5 weeks (range, 0 to 180) in the placebo group. Patients who experienced a relapse in the double-blind period or those who completed the double-blind period without any relapse could enter the open-label extension period wherein they could receive satralizumab s.c. at weeks 0, 2, and 4 and monthly thereafter for 1 year (depending on the condition) in combination with a baseline treatment or as a monotherapy. The median duration of treatment among patients who received satralizumab in the double-blind and open-label extension periods was 143.1 weeks (range, 15 to 224)

^b^Benign neoplasm of the thyroid gland, colon adenoma, and uterine leiomyoma occurred in one patient each in the anti–IL-6 therapy group

*AQP4-Ab* aquaporin-4 antibody, *AZA* azathioprine, *CI* confidence interval, *CR* case report, *CRP* C-reactive protein, *CS* case series, *CT* phase 3, double-blind, randomized, placebo-controlled trial, *CyA* cyclosporine, *IQR* interquartile range, *IRR* injection-related reaction, *i.v.* intravenous, *MMF* mycophenolate mofetil, *NMO* neuromyelitis optica, *NMOSD* neuromyelitis optica spectrum disorder, *NRS* numerical rating scale, *PSL* prednisolone, *ROS* retrospective observational study, *SBP* systolic blood pressure, *s.c.* subcutaneous, *SD* standard deviation, *SEM* standard error of the mean, *SLE* systemic lupus erythematosus, *VAS* visual analog scale

## IL-6 signaling in CNS

**Fig. 1:**
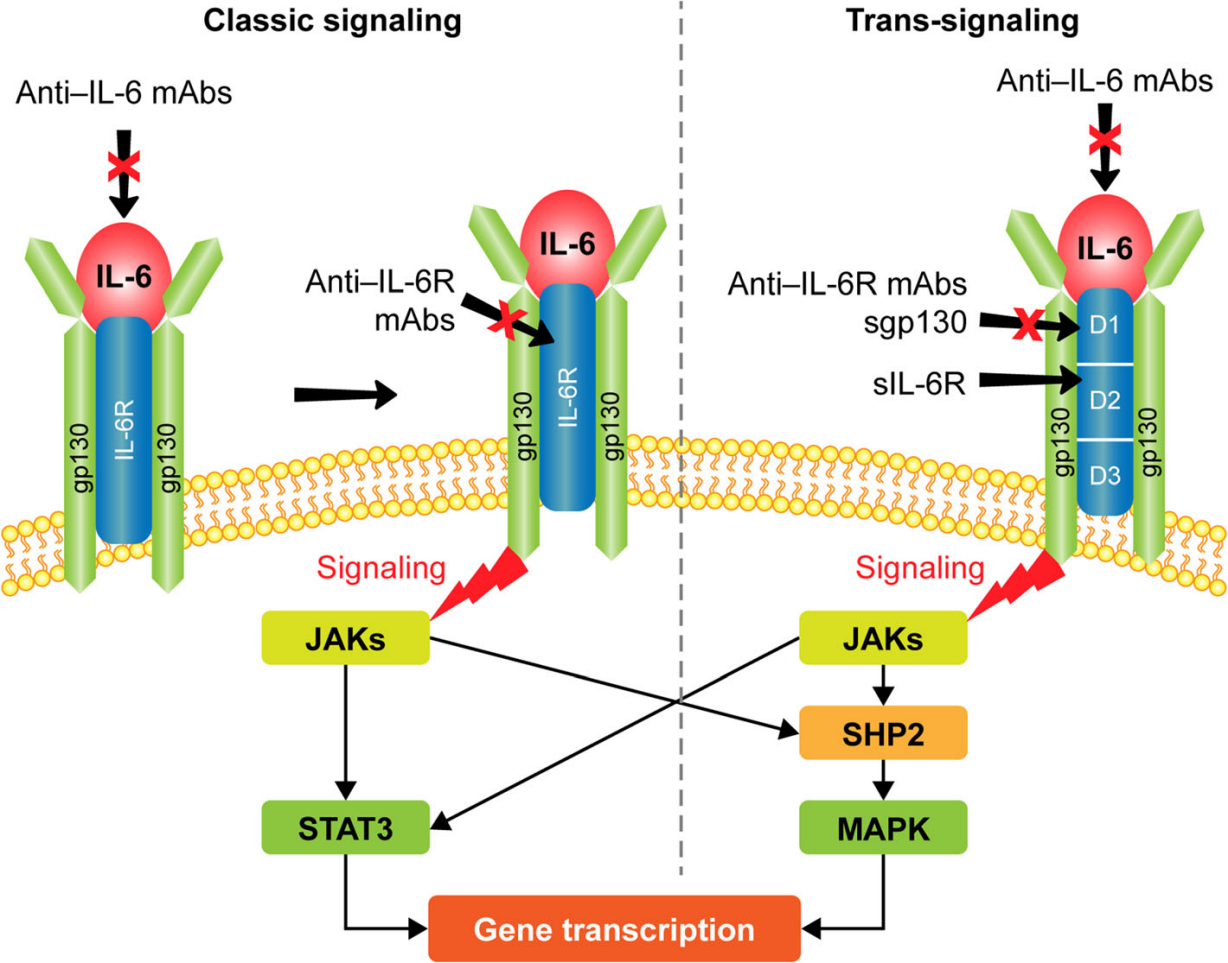
IL-6 signaling. D1–D3, subdomains of IL-6Rα; gp130, glycoprotein 130; IL-6, interleukin-6; IL-6R, interleukin-6 receptor; JAK, Janus kinase; mAb, monoclonal antibody; MAPK, mitogen-activated protein kinase; sgp, soluble glycoprotein 130; SHP2, Src homology region 2 domain–containing phosphatase-2; sIL-6R, soluble IL-6R; STAT3, signal transducer and activator of transcription 3

Neurons, astrocytes, microglia, and endothelial cells are essential sources of IL-6 in the CNS [28]. Under physiological conditions, all of them may produce small amounts of IL-6, but stimuli, such as an injury, trigger the release of large amounts of IL-6 [28], which can bind either to the membrane bound (classic signaling) or the soluble form (trans-signaling) of the IL-6R [29]. The IL-6/IL-6R complex subsequently binds to a second receptor subunit, glycoprotein 130 (gp130), which dimerizes and induces intracellular downstream signaling [29]. This leads to the activation of several signal transduction pathways including the Janus kinase (JAK)/signal transducer and activator of transcription (STAT), mitogen-activated protein kinase (MAPK), phosphoinositide 3-kinase (PI3K), and AKT pathways, via five tyrosine residues within the cytoplasmic portion of gp130 [29]. While classic signaling may be blocked by anti–IL-6 and anti–IL-6R antibodies, trans-signaling may be blocked by both anti–IL-6 and IL-6R antibodies and the soluble form of gp130 (Fig. 1) [30]. In addition to trans-signaling via the soluble IL-6R, IL-6R is expressed on both oligodendrocyte progenitor cells and microglia; therefore, IL-6 signaling in the CNS may have both direct and indirect effects on microglial and macroglial survival [31].

## Role of IL-6 in the pathogenesis of MS and NMOSD

**Fig. 2:**
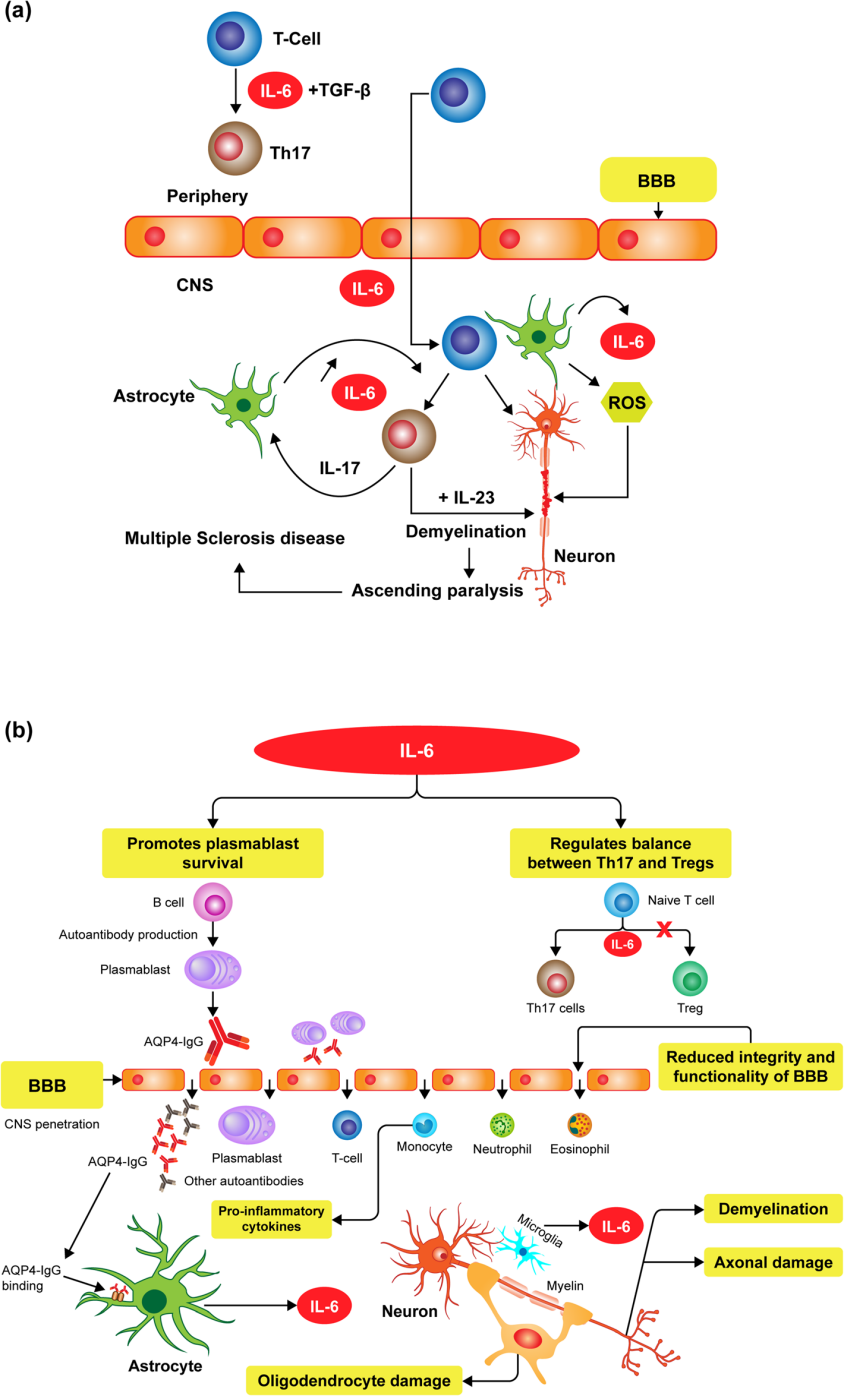
**A** Role of IL-6 in the pathophysiology of multiple sclerosis. BBB, blood–brain barrier; CNS, central nervous system; IL, interleukin; ROS, reactive oxygen species; TGF-β, transforming growth factor beta; Th, T-helper. **B** Role of IL-6 in the pathophysiology of NMOSD. AQP4, aquaporin-4; BBB, blood–brain barrier; CNS, central nervous system; IgG, immunoglobulin G; IL-6, interleukin 6; NMOSD, neuromyelitis optica spectrum disorder; Th, T helper; Treg, regulatory T cell

Myelin-specific cluster of differentiation (CD)4+ T cells play a central role in the pathogenesis of MS [32]. CD4+ T cells, activated in the periphery, infiltrate the CNS and initiate an inflammatory cascade by secreting chemokines and cytokines [32]. In turn, the release of IL-6 enhances T-cell function by inducing their proliferation and infiltration into the CNS through upregulation of vascular cell adhesion molecule 1 on the vascular endothelial cells (Fig. 2A). In the presence of transforming growth factor-β, IL-6 also induces the differentiation of naïve CD4+ T cells into inflammatory T-helper (Th) 17 cells, which secrete IL-17 and thus stimulate the production of IL-6 in astrocytes via a positive feedback loop [28]. IL-6, reactive oxygen species, and nitric oxide are produced in astrocytes after induction facilitated by direct contact with T cells, which further damage the oligodendrocyte myelin sheath, thereby leading to ascending paralysis and, in the presence of IL-23, resulting in the full development of MS [28]. Maimone et al. demonstrated IL-6 immunoreactivity in MS lesions representative of acute and chronic active plaques from brains of six patients with secondary progressive MS [33]. In addition, IL-6 was more frequently detected in the cerebrospinal fluid (CSF) of patients with MS compared with patients with other noninflammatory neurological diseases [34].

There is growing evidence for the role of the IL-6 signaling pathway in NMOSD pathogenesis [31]. In B cells isolated from AQP4-IgG seropositive patients with NMOSD, IL-6 promoted plasmablast survival and stimulated AQP4-IgG secretion, whereas blockade of IL-6 signaling by an anti–IL-6R antibody reduced the survival of plasmablasts in vitro [35]. These findings suggest that an IL-6–dependent B-cell subpopulation is involved in the pathogenesis of NMOSD, thereby providing a therapeutic strategy for targeting the IL-6R signaling pathway [35]. IL-6 also reduces the integrity and functionality of the blood–brain barrier (BBB) and enhances the differentiation and activation of T-lymphocytes [31, 35]. Furthermore, IL-6 maintains homeostasis of the immune system by regulating the balance between Th17 and regulatory T cells (Tregs) (Fig. 2B); an imbalance between Th17 and Tregs may contribute to autoimmunity [36]. Increased levels of IL-6 have been reported in the serum and CSF of patients with NMOSD [37, 38]. Furthermore, IL-6 levels in the CSF positively correlated with AQP4-IgG and glial fibrillary acidic protein levels, an indicator of astrocyte damage [38].

## Role of IL-6 in common animal models of neuropathic pain

Messenger ribonucleic acid (mRNA) levels of IL-6, a pro-inflammatory cytokine, are elevated in the spinal cord of rats with neuropathic pain [39]. IL-6 may induce neuropathic pain directly by nociceptive effect or indirectly by stimulating the production of pain mediators, such as prostaglandin [40, 41]. Intrathecal administration of IL-6 produced touch-evoked allodynia in normal rats and thermal hyperalgesia in rats with sciatic cryoneurolysis, which is used as an animal model of neuropathic pain [40]. Furthermore, intrathecal administration of a neutralizing IL-6 antibody significantly attenuated mechanical allodynia following peripheral nerve injury in rats [42]. Microinjection of recombinant human IL-6 into the lateral cerebroventricle in rats reduced the paw withdrawal latency as evaluated by the hot plate test, indicating the hyperalgesic effect of IL-6 [43]. In contrast, the paw withdrawal latency was increased when sodium salicylate, an inhibitor of prostanoid biosynthesis, was co-injected with IL-6 [43]. This study demonstrated the prostanoid-dependent indirect nociceptive effect of IL-6 [43].

IL-6 plays a role in mediating neuropathic pain associated with cancer, peripheral nerve injury, spinal cord injury, chemotherapy-induced peripheral neuropathy, and inflammatory pain [44]. Although the pathogenesis of neuropathic pain in neuroimmunological disorders is complex, the JAK/STAT3 and MAPK signaling pathways induced by IL-6 could be the common underlying pathways [44]. In rats with chronic constriction injury (CCI) of the sciatic nerve, which is an animal model of peripheral nerve injury, the protein levels of IL-6 in the ipsilateral dorsal horn were significantly increased compared with naïve control rats [45]. IL-6 induced the expression of microglial CX3CR1 in the spinal cord through activation of p38 MAPK, thereby enhancing the responsiveness of microglia to fractalkine in the spinal cord [45]. Furthermore, intrathecal administration of an anti-CX3CR1 neutralizing antibody attenuated the mechanical allodynia and thermal hyperalgesia induced by recombinant IL-6 in naïve rats [45]. In another study, the increased levels of IL-6 in the spinal cord led to rapid activation of the JAK/STAT3 signal transduction pathway in the spinal cord microglia [46]. The blockade of JAK/STAT3 signaling by SOCS3, a physiologic inhibitory protein of JAK/STAT3, markedly attenuated the development of mechanical allodynia, which is a characteristic feature of CCI-induced neuropathic pain [46]. Chemotherapy-induced neuropathic pain due to IL-6 may also be attributed to the activation of the JAK pathway, which then triggers downstream signaling of PI3K and transient receptor potential vanilloid channel type 1 [44].

## Role of IL-6 in neuropathic pain of experimental autoimmune encephalomyelitis

Experimental autoimmune encephalomyelitis (EAE) is a widely used animal model for MS, and EAE mice exhibit many features observed in patients with MS, including human pain reactions [12]. EAE is induced by immunization with myelin components such as myelin oligodendrocyte glycoprotein (MOG) and is characterized by demyelination, axonal damage, recruitment of T cells and other innate immune cells, glial cell activation, and pro-inflammatory cytokine and chemokine signaling [12]. Cold and tactile allodynia are the predominant sensory disturbances in the MOG EAE model, develop prior to any signs of overt neurological dysfunction associated with MS, and are attributed to the enhanced reactivity of microglia and an increased presence of CD3+ T cells in the superficial dorsal horn of mice [47].

IL-6-deficient mice are resistant to MOG-induced EAE compared with wild-type mice with delayed-type hypersensitivity response, lymphocyte proliferation response, and antibody reactivity to MOG in IL-6-deficient mice being significantly lower than those in wild-type mice [48]. IL-6 has been hypothesized to play a crucial role in the induction phase of EAE based on research showing that IL-6-deficient mice immunized with MOG developed milder EAE and at a significantly lower frequency when compared with wild-type mice; however, those injected with IL-6 before the onset of motor dysfunction showed a significantly delayed onset, but developed severe EAE at a high frequency [49]. In addition, the serum concentrations of Th1 and Th17 cytokines, IL-6, IL-1β, IL-1α, and prostaglandin E2 in EAE mice were significantly higher than those observed in control mice [50]. Furthermore, a statistically significant positive correlation was found between the IL-6/IL-10 ratio and EAE severity, demyelination rate, and lymphocyte infiltration in EAE mice [50]. In addition, nuclear factor kappa B triggers a positive feedback loop for IL-6 expression (IL-6 amplifier) in endothelial cells, and its activation can lead to excess expression of various chemokines and cytokines including CCL20 and IL-6 and the development of autoimmune diseases such as EAE [51].

Our research group has evaluated the efficacy of an intraperitoneal injection of MR16-1, a rat anti–mouse IL-6R antibody, on pain sensitivity in EAE mice [52, 53]. EAE was induced by subcutaneous administration of MOG_35–55_ (200 μL of an emulsion containing MOG_35–55_ in complete Freund’s adjuvant supplemented with *Mycobacterium tuberculosis* extract H37Ra) on day 0 followed by intravenous administration of 300 ng of pertussis toxin on days 0 and 2 [52]. MR16-1 was administered to the mice around the time of MOG injection (day 0 or 3; experiment 1) and on day 12 after MOG injection (experiment 2) when significant pain had developed [52]. Mechanical allodynia was evaluated as the paw withdrawal threshold using calibrated von Frey filaments (force, 0.04–2.0 g) before immunization with MOG, and on days 7, 14, and 20 in experiment 1 and before immunization, on day 12, and after EAE onset in experiment 2 [52]. In experiment 1, MR16-1 administered on days 0 or day 3 significantly reduced mechanical allodynia at days 7, 14, and 20 and prevented the induction of motor dysfunction in EAE mice [52]. In experiment 2, MR16-1 significantly increased the paw withdrawal threshold at EAE onset; however, the clinical score (paralysis score) was approximately equal between mice that received the control vehicle and those that received MR16-1 [52]. The CSF/serum ratio of MR16-1 concentration 2 days after administration was greater in EAE mice than in normal mice, suggesting the passage of MR16-1 through the BBB [52]. The antinociceptive effect of MR16-1 on spontaneous pain in EAE mice was also evaluated using a standardized murine facial expression-based coding system, the Mouse Grimace Scale (MGS) [53]. EAE was induced by subcutaneous administration of 50 μg MOG emulsified in complete Freund’s adjuvant (CFA) supplemented with *Mycobacterium tuberculosis* on day 0 and 250 ng of pertussis toxin (intravenously on day 0 and intraperitoneally on day 2) [53]. Control mice received CFA and saline [53]. The MGS score in EAE mice on day 19 was significantly higher compared with that in control mice [53]. Administration of MR16-1 also prevented an increase in the MGS score, especially in the pre-onset phase [53]. Facial grimacing in EAE mice could be influenced by the demyelination of lower motor neurons, which results in brainstem reflex abnormalities and a compromised ability to exhibit facial expressions [54]. Thus, the increased MGS scores in post-onset EAE mice may be attributed to factors other than spontaneous pain and the inability to correctly evaluate MGS [53]. However, the significant improvement in the levels of spinal serotonin (5-HT) and the ratio of 5-hydroxyindoleacetic acid (the major metabolite of 5-HT) to 5-HT (5-HIAA/5-HT) with MR16-1 in post-onset EAE mice suggest that MR16-1 may have had an effect on spontaneous pain in these mice [53]. Further investigations are needed to elucidate the effect of anti–IL-6R antibodies on spontaneous pain in post-onset EAE mice.

## Mechanisms of anti–IL-6 therapies for neuropathic pain in EAE

### Microglial activation

Microglial cells are quiescent immune cells of the CNS that play an important role in initiating, sustaining, and mediating neuropathic pain [55]. Upon activation, microglial cells develop the ability to phagocytose, present antigens to T lymphocytes and release cytokines [55]. Following nerve injury, molecules released from neurons contribute to microglial activation, causing changes in their morphology, migration to the site of injury, and increased proliferation through a process called microgliosis [55]. Microglial cells are activated following peripheral nerve injury and remain active for several weeks [56]. Activated microglial cells start producing a number of cytokines such as IL-6, which plays an important role in the development of neuropathic pain [56]. Nerve injury–induced microgliosis is also associated with the development of pain hypersensitivity [57]. Reduction in microglial activation has been shown to ameliorate neuropathic pain in diabetic neuropathy and CCI rat models [58, 59]. Decreased mechanical allodynia by MR16-1 in EAE mice was also attributed to the inhibition of microglial activation and proliferation in the spinal cord [52]. IL-6 activates microglial JAK/STAT3 signaling in the spinal cord after peripheral nerve injury [60]. Phosphorylation of STAT3 was significantly increased in the spinal cord of EAE mice and was significantly decreased by administration of MR16-1 [52].

### Descending pain inhibitory pathway

The descending pain inhibitory pathway originates from various sites in the brainstem, most notably the midbrain periaqueductal gray (PAG) and the medullary raphe nuclei, descends via the dorsolateral funiculus to all levels of the spinal cord, and plays an important role in adequate spinal nociception [15]. The descending pathways exhibit dramatic plasticity and multiplicity and are actively involved in the development and maintenance of persistent pain after tissue or nerve injury through their contribution to altered pain processing after injury [61]. In patients with NMOSD, descending inhibition could be impaired by lesions at the origin of the descending pathway and/or along the descending fiber tracts [15]. Periaqueductal lesions are relatively frequent and found during all clinical stages in patients with MS [62] and in patients with AQP4-IgG seropositive NMOSD [63], suggesting an impaired descending pain inhibitory system. NMOSD lesions are most frequently found in the gray matter of the cervical spinal cord [15]. Extensive NMOSD lesions that reach the nearby white matter could also affect the spinal fiber tracts, including those comprising the descending inhibitory pathways, and the functioning of the descending antinociceptive systems, thereby causing severe spontaneous pain and hyperalgesia at and below the vertebral levels of the lesions [15]. In EAE mice that received MR16-1, the blood oxygenation level–dependent (BOLD) responses were assessed using functional magnetic resonance imaging (fMRI) after the application of mechanical stimuli (von Frey filaments) to the pad of the right limb of the mice [53]. The BOLD signal intensity in the PAG after the application of mechanical stimuli was significantly lower in pre-onset EAE mice than in control mice, suggesting that the descending pain inhibitory system was impaired in EAE mice [53]. MR16-1–treated mice showed a higher mean value of percentage change in signal intensity compared with vehicle-treated mice [53]; however, the change was not significant due to a large intragroup variation. Thus, administration of an anti–IL-6R antibody such as MR16-1 had the potential to protect against spontaneous pain in EAE mice which was partly caused by impairment in the descending pain inhibitory system [53].

## Future directions

Anti–IL-6 therapies have shown evidence in the treatment of neuropathic pain in EAE mice. Screening of anti–IL-6 therapies in NMOSD-specific models could help provide further insights into their mechanism(s) of action. Evaluation of the effects of anti–IL-6 therapies in ameliorating neuropathic pain as a primary endpoint in large clinical trials is warranted and may help address the unmet medical need in the management of neuropathic pain associated with neuroimmunological disorders such as MS and NMOSD. It is important to design trials specifically to evaluate the effect of anti–IL-6 therapies on pain in patients with neuroimmunological disorders with suitable objective measures of pain and without confounding factors such as concomitant use of analgesics. The effect of anti–IL-6 therapies may also be influenced by the baseline severity of neuropathic pain in patients and it may be prudent to understand the effect of anti–IL-6 therapies in patients with varying degrees of pain severity.

## Conclusions

**Fig. 3:**
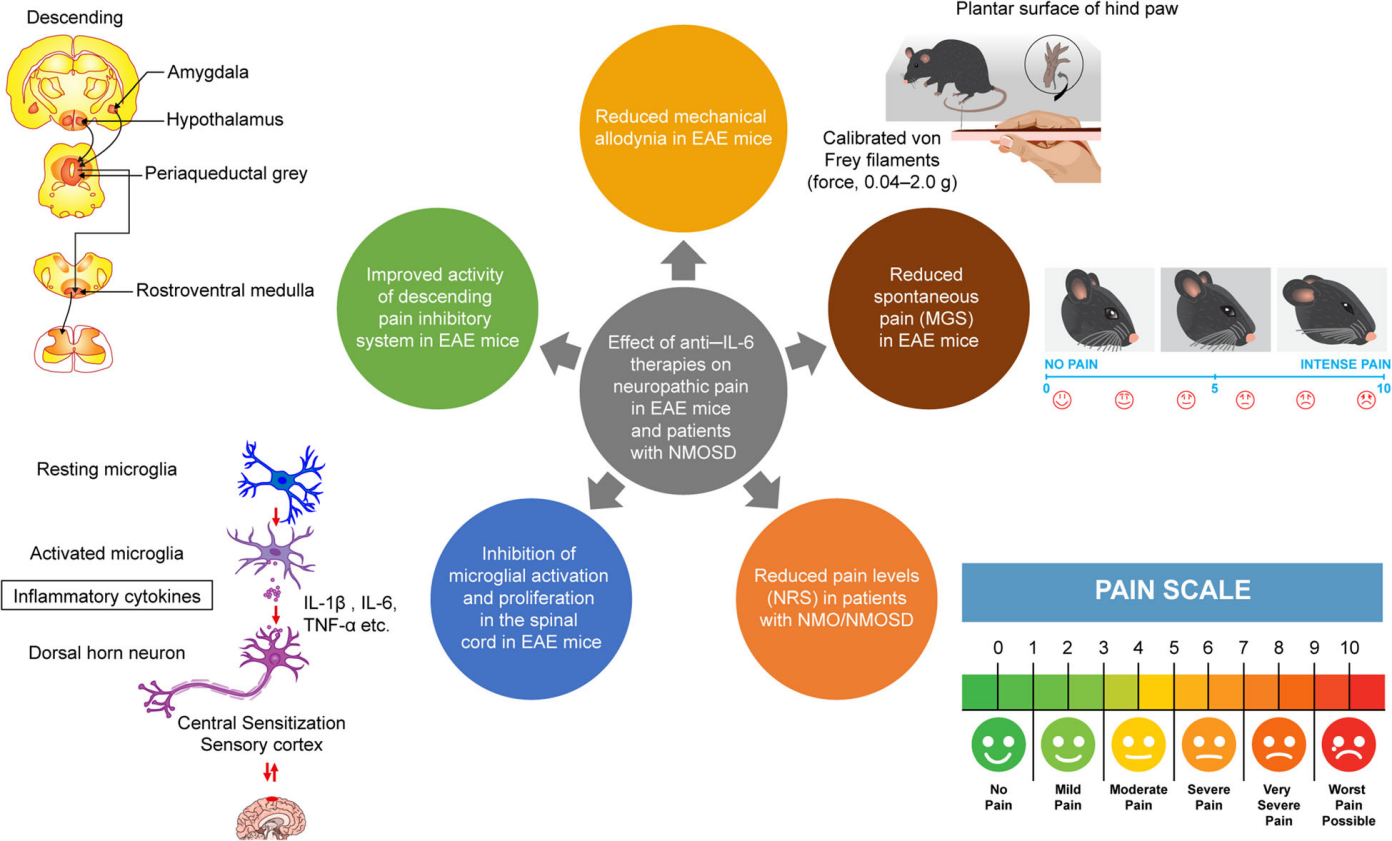
Effects of anti–IL-6 therapies on neuropathic pain in preclinical and clinical studies. EAE, experimental autoimmune encephalomyelitis; IL, interleukin; MGS, Mouse Grimace Scale; MS, multiple sclerosis; NMO, neuromyelitis optica; NMOSD, neuromyelitis optica spectrum disorder; NRS, numerical rating scale; TNF-α, tumor necrosis factor alpha

There is an unmet medical need for the treatment of neuropathic pain in patients with neuroimmunological disorders. Current treatments for neuropathic pain such as pregabalin and gabapentin, TCAs, and SSNRIs do not target the underlying mechanisms causing pain and are not specific to the different phases of the development of neuropathic pain. IL-6 levels are increased in the CSF of patients with MS and NMOSD, and it is believed to have a role in the pathophysiology of both conditions. Recent preclinical studies of MR16-1, an anti–IL-6R antibody, have demonstrated amelioration of mechanical allodynia and spontaneous neuropathic pain in EAE mice through the inhibition of microglial activation and the descending pain inhibitory system. The effects of anti–IL-6 therapies in the management of neuropathic pain associated with MS and NMOSD are summarized in Fig. 3. Further clinical and non-clinical investigations are needed to establish IL-6 therapy as a target for the treatment of neuropathic pain associated with neuroimmunological disorders such as MS and NMOSD.

## Data Availability

Not applicable

## Abbreviations

5-HT: Serotonin
5-HIAA: 5-Hydroxyindoleacetic acid
AQP4: Aquaporin-4
AZA: Azathioprine
BBB: Blood–brain barrier
BOLD: Blood oxygenation level–dependent
CCI: Chronic constriction injury
CD: Cluster of differentiation
CFA: Complete Freund’s adjuvant
CI: Confidence interval
CNS: Central nervous system
CR: Case report
CRP: C-reactive protein
CS: Case series
CSF: Cerebrospinal fluid
CX3CR1: C-X3-C chemokine receptor type 1
CyA: Cyclosporine
EAE: Experimental autoimmune encephalomyelitis
fMRI: Functional magnetic resonance imaging
gp130: Glycoprotein 130
i.v.: Intravenous
IgG: Immunoglobulin G
IL: Interleukin
IL-6R: Interleukin-6 receptor
IRR: Injection-related reaction
JAK: Janus kinase
MAPK: Mitogen-activated protein kinase
MOG: Myelin oligodendrocyte glycoprotein
MMF: Mycophenolate mofetil
MGS: Mouse Grimace Scale
mRNA: Messenger ribonucleic acid
MS: Multiple sclerosis
NMO: Neuromyelitis optica
NMOSD: Neuromyelitis optica spectrum disorder
NRS: Numeric rating scale
PAG: Periaqueductal gray
PI3K: Phosphoinositide 3-kinase
PSL: Prednisolone
SD: Standard deviation
sgp130: Soluble glycoprotein 130
SOCS3: Suppressor of cytokine signaling 3
SSNRI: Selective serotonin-norepinephrine reuptake inhibitor
STAT: Signal transducer and activator of transcription
TCA: Tricyclic antidepressant
TGF-β: Transforming growth factor beta
Th: T-helper
TNF-α: Tumor necrosis factor alpha
Treg: Regulatory T cell
VAS: Visual analog scale
